# αν and β1 Integrins Mediate Aβ-Induced Neurotoxicity in Hippocampal Neurons via the FAK Signaling Pathway

**DOI:** 10.1371/journal.pone.0064839

**Published:** 2013-06-03

**Authors:** Hai-Yan Han, Jin-Ping Zhang, Su-Qiong Ji, Qi-Ming Liang, Hui-Cong Kang, Rong-Hua Tang, Sui-Qiang Zhu, Zheng Xue

**Affiliations:** 1 Department of Neurology, Tongji Hospital, Huazhong University of Science and Technology, Wuhan, Hubei Province, China; 2 Department of Neurology, Qianfoshan Hospital, Shan Dong University, Jinan, Shandong Province, China; National Institute on Aging Intramural Research Program, United States of America

## Abstract

αν and β1 integrins mediate Aβ–induced neurotoxicity in primary hippocampal neurons. We treated hippocampal neurons with 2.5 µg/mL 17E6 and 5 µg/mL ab58524, which are specific αν and β1 integrin antagonists, respectively, for 42 h prior to 10 µM Aβ treatment. Next, we employed small interfering RNA (siRNA) to silence focal adhesion kinase (FAK), a downstream target gene of integrins. The siRNAs were designed with a target sequence, an MOI of 10 and the addition of 5 µg/mL polybrene. Under these conditions, the neurons were transfected and the apoptosis of different cell types was detected. Moreover, we used real-time PCR and Western blotting analyses to detect the expression of FAK and ρFAK genes in different cell types and investigated the underlying mechanism and signal pathway by which αν and β1 integrins mediate Aβ-induced neurotoxicity in hippocampal neurons. An MTT assay showed that both 17E6 and ab58524 significantly increased cell viability compared with the Aβ-treated neurons (*P*<0.01 and *P*<0.05, respectively). However, this protective effect was markedly attenuated after transfection with silencing FAK (siFAK). Moreover, TUNEL immunostaining and flow cytometry indicated that both 17E6 and ab58524 significantly protected hippocampal neurons against apoptosis induced by Aβ (*P*<0.05) compared with the Aβ-treated cells. However, this protective effect was reversed with siFAK treatment. Both the gene and protein expression of FAK increased after Aβ treatment. Interestingly, as the gene and protein levels of FAK decreased, the ρFAK protein expression markedly increased. Furthermore, both the gene and protein expression of FAK and ρFAK were significantly diminished. Thus, we concluded that both αν and β1 integrins interfered with Aβ-induced neurotoxicity in hippocampal neurons and that this mechanism partially contributes to the activation of the Integrin-FAK signaling pathway.

## Introduction

Alzheimer’s disease (AD), the most common type of dementia, is an aged-associated neurodegenerative disease caused by complicated interactions between genetic and environment factors [1]. It is currently without effective disease modifying treatment [2]. Between 2000 and 2008, the population of deaths due to heart disease, stroke, and prostate cancer decreased by 13%, 20%, and 8%, respectively, whereas the population due to AD increased by 66% [3]. Three major pathological features are extracellular abnormal deposition of β-amyloid (Aβ), the formation of senile plaques by glial cell activation, and the formation of neurofibrillar tangles by the aberrant phosphorylation of the intracellular protein Tau [4], [5]. These pathologies result in dysfunction and neuronal loss in the hippocampus and cortex in AD patients [6]. Several previous studies have demonstrated that abnormal formation and deposition of Aβ are extremely critical to AD development [7], [8]. Extracellular studies have shown that the fibrillation of Aβ promotes the loss and apoptosis of neurons [5], [9]; however, the underlying mechanism is still unknown. A more recent study has told that Aβ derives from amyloid precursor protein(APP) [10]. Thus we propose that Aβ promotes neurotoxicity, which affects specific neuronal mediators localized to the membrane and their interactions. It has been shown that several receptors and proteins interact with Aβ [11].

The integrin (Itg) family is a class of cellular adhesion molecules with adhesive and signal-transduction functions. They function as heterodimers, which consist of α and β subunits, and bind non-covalently to mediate cell-cell and cell-extracellular matrix interactions. Itgs participate in the regulation of the signaling pathways involved in growth, hypertrophy, survival, differentiation, migration, cell morphology and apoptosis [12], [13], [14], [15]. Growing evidence has demonstrated that Itgs play an important role in AD. Hetero-αν antagonists can block the inhibition of long-term potentiation induced by Aβ in vivo and in vitro, suggesting that the αν Itg is an important regulator of synaptic dysfunction, plasticity diversity and long-term potentiation in the early stages of neurodegenerative diseases [16]. The heterodimers α2β1, α5β1, ανβ1 and ανβ3 can facilitate the deposition of Aβ and induce neurotoxicity, which results in neuronal loss [17], [18], [19]. However, the mechanism by which AD is mediated by Itgs is still unknown.

Our study used cultured primary hippocampal neurons as a model for Aβ neurotoxicity. We used the specific antagonists, αν and β1, to observe the function of these two Itg subunits during Aβ-induced apoptosis. Using siRNA technology, focal adhesion kinase (FAK), an important downstream effector of Itgs, was silenced to explore the mechanism of αν- and β1-mediated Aβ-induced neurotoxicity. We found that both 17E6 and ab58524, which are specific antagonists of αν and β1, respectively, inhibited the apoptosis of hippocampal neurons induced by Aβ, indicating a potential link with the activation of the Itg-FAK signaling pathway.

## Materials and Methods

### Ethics Statement

All procedures were approved by the Committee on the Ethics of Animal Experiments of Huazhong University of Science and Technology and were in strict accordance with the recommendation in the Guide for the Ethical Issues in Animal Experimentation.

We dissected out the hippocampi of neonatal rats at postnatal day 1 (P1) and cultivated the neurons in vitro. We used microtubule associated protein-2 (MAP-2) immunostaining to examine the purity of the neuronal population. Fibrillary Aβ at different concentrations was administered to the cells at different time points. A MTT colorimetry assay was used to test for cell activity and determine the most optimal time and concentration to establish the Aβ neurotoxicity model in vitro.

We designed three target siRNA sequences (S1, S2 and S3) against the FAK gene *PtK2* in rat hippocampal neurons. Following enzyme digestion, transformation, PCR assessment, positive clone sequencing and lentiviral assembly, the viral concentration was assayed. Fluorescence microscopy was used to observe the cellular GFP expression in different cell types and assay the transfection efficiency of the lentivirus under different conditions. Real-time PCR and Western blotting analyses were used to detect the FAK gene and protein expression, respectively.

Prior to the 10 µM Aβ treatment, the cells were administered 2.5 µg/mL and 5 µg/mL 17E6 and ab58524, which are specific antagonists of Itg αν and β1, respectively, for 42 h. Next, MTT colorimetry, flow cytometry and TUNEL staining were performed to examine neuronal apoptosis.

To silence FAK, siRNA technology was used with the optimal silencing target sequence T1 (the MOI was 10) and the addition of 5 µg/ml polybrene, which enhanced the transfection efficiency. Silencing FAK (siFAK) was used to interfere with the function of different cell types and examine neuronal apoptosis.

Using real-time PCR and western blotting techniques, we examined the gene and protein expression of FAK and ρFAK, respectively, in different cell types to understand the mechanism and contribution of the αν and β1-mediated apoptotic signaling pathway in Aβ-induced neurotoxicity in cultured hippocampal neurons.

## Results

### MAP-2 Detects Neuronal Purity in the Hippocampus

Immunostaining with the neuron-specific marker MAP-2 was performed to visualize and determine the neuronal purity in primary hippocampal neurons (greater than 95%) (Image-Pro Plus 6.0 software). The hippocampal neurons were immunolabeled using MAP-2 and DAPI (Figure S1).

### Build-up Neurotoxicity Model

**Figure 1:**
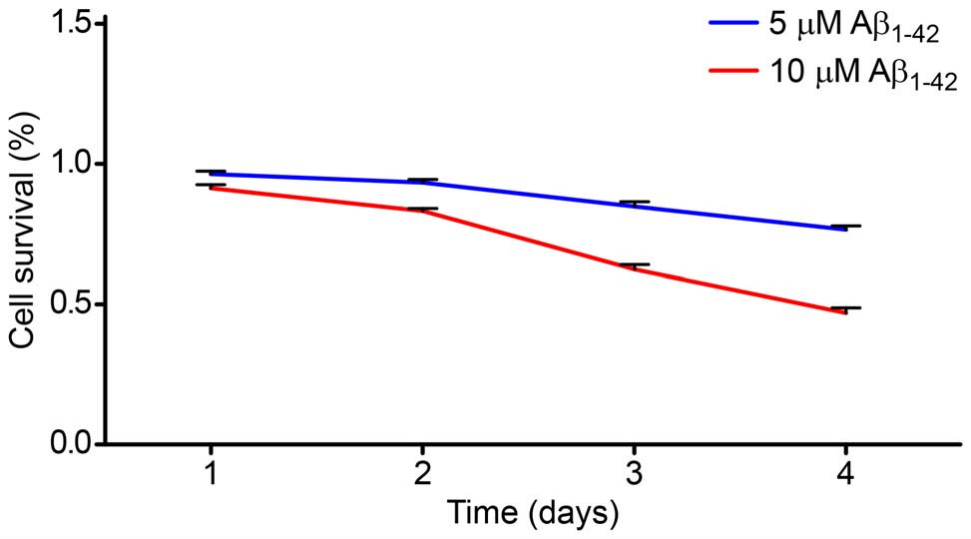
MTT assay detection of the survival rate of hippocampal neurons.

We measured the survival rate of hippocampal neurons at different time points and concentrations with aging Aβ fibrosis using MTT colorimetry. Aβ-induced neurotoxicity demonstrated a temporal- and concentration-dependence in hippocampal neurons in vitro (Figure 1). The survival rate of the neurons was 62.3% compared with the control group after treatment with 10 µM Aβ for three days. After four days of treatment, the survival rate decreased to 46.9%. We used siRNA technology to silence the integrin downstream target protein FAK and further explored the contribution of αν and β1 integrins in Aβ neurotoxic pathology. We adopted a three-day treatment as a model to examine the apoptosis of hippocampal neurons.

### Build-up Carriers

We successfully prepared dsDNA and Hpa I/Xho I enzyme-digested-linearized pFU-GW-iRNA carriers (Figure S2). And we ligated and transformed the construct into competent cells (Figure S3). The positive clone colony PCR segments were 343 bp, and the vshRNA-n on-connected empty carrier clone PCR segments were 299 bp. The ddH_2_O negative control group contained no PCR segments. The selective carriers that contained the target fragments completely corresponded with the design sequence after sequencing appraisal using Chromas software.

### Measurement of the Lentiviral Titers

Following viral packaging and concentration determination, the *PtK2* RNA-interfering (RNAi) lentiviral carriers were transfected into 293T cells as previously described. After four days, GFP expression was observed using an inverted fluorescence microscope. A dilution titer method was used to detect the GFP expression in cells transfected with different concentrations of viral solution, and the biological titers of the viral solution were calculated for these three sequences (S1, S2 and S3), which were 6×108, 8×108 and 6×108 TU/mL, respectively (Figure S4).

### The Most Optimal Lentiviral Transfection Conditions and Infection Efficiency in Hippocampal Neurons in vitro

Based on the GFP expression in hippocampal neurons, as visualized using immunofluorescence microscopy, the transfection efficiency in hippocampal neurons of three lentiviral carriers (S1, S2, S3) in four different MOIs (0, 1, 5, 10) with or without the addition of the transfection enhancer polybrene (5 µg/mL) may be calculated (Figure S5). The transfection efficiency increased with the increase in polybrene and MOI. When the MOI was 10 and in the presence of polybrene, the lentiviral transfection efficiency was enhanced by 90%. These conditions were used in the following experiments.

### FAK Gene Expression after Lentiviral Transfection in Hippocampal Neurons

When the MOI was 10 and in the presence of polybrene, the three siRNA sequences blocked *PtK2* gene expression in hippocampal neurons after 72 h of transfection. If the *PtK2* expression in the control group was 1, the relative expression in S1, S2 and S3 was 0.346±0.013, 0.546±0.099 and 0.648±0.049, respectively, in the hippocampal neurons after transfection. The *PtK2* expression in S1 and S2 exhibited statistically significant heterogeneity (P<0.05 or P<0.01) (Figure S6).

### FAK Protein Expression after Lentiviral Transfection in Hippocampal Neurons

When the MOI was 10 and in the presence of polybrene, the three siRNA sequences silenced the FAK protein expression in hippocampal neurons after 72 hours of transfection. If the FAK protein expression in the control group was 1, the relative expression in S1, S2 and S3 was 0.514±0.098, 0.659±0.343 and 0.659±0.343, respectively, in hippocampal neurons after transfection. The FAK protein expression in S1 and S2 exhibited statistically significant heterogeneity (both P<0.05) (Figure S7).

### Treatment with 17E6 and ab58524 Enhanced the Survival Rate in Hippocampal Neurons Induced by Aβ

**Figure 2:**
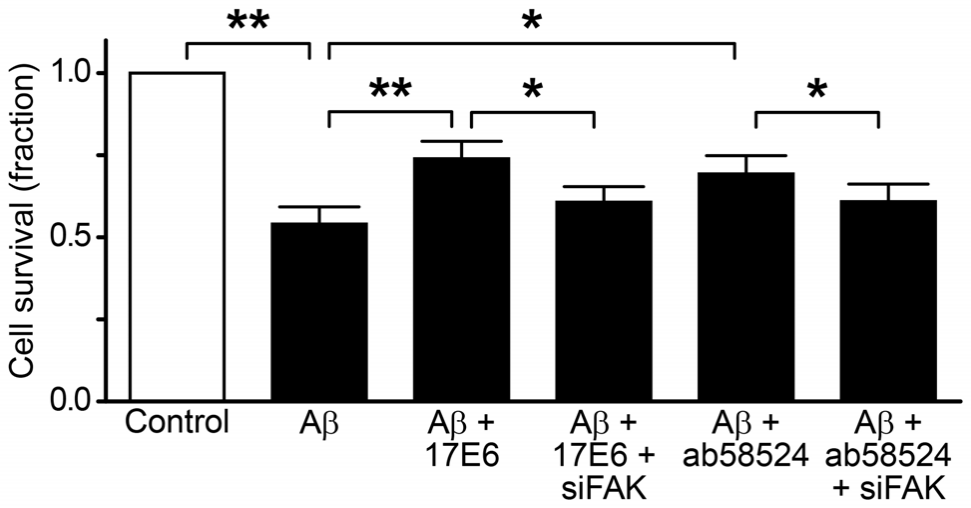
The survival rate after MTT assay; **P<0.01, *P<0.05.

The MTT assay examines cell activity on the basis of the absorbance diversity. In contrast with the control group, the MTT results showed that the cell survival rate decreased to 0.54±0.12 after treatment with 10 µM Aβ in hippocampal neurons for 72 h (Figure 2). However, the survival rate increased after pretreating the cells with 2.5 µg/mL 17E6 and 5 µg/mL ab58524, which are Itg αν and β1 antagonists, respectively. The survival rates of 17E6 and ab58524 were 0.74±0.13 and 0.70±0.13, respectively, and were significantly different from the Aβ treatment group (P<0.01 and P<0.05, respectively). Although 17E6 and ab58524 enhanced the neuronal survival rate, there was still a statistically significant difference compared with the control group (P<0.05).

### 17E6 and ab58524 Inhibited the Apoptosis of Hippocampal Neurons Induced by Aβ

**Figure 3:**
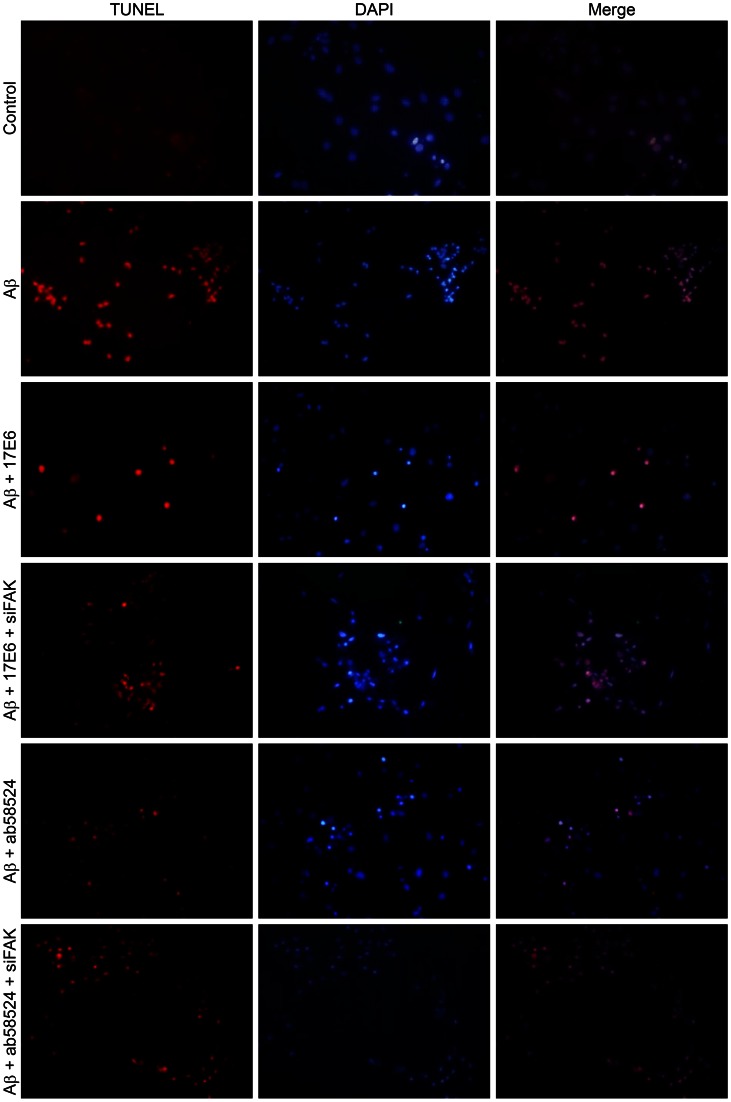
Detection of the apoptotic rate by TUNEL staining (×200).

The TUNEL assay identifies in situ the integral single apoptotic nuclei or apoptotic body, which accurately reflects the biochemical and morphological characteristics of apoptosis. The TUNEL assay exhibits very high sensitivity and may uncover a very small number of apoptotic cells. For this reason, this method may be widely used in apoptotic studies. As illustrated in Figure 3, there were very few apoptotic cells in the control group compared with the large number of TUNEL-positive cells in the Aβ treatment group. Chromatin condensation occurs in the presence of apoptosis. DAPI staining produces a dense stain in the nuclei and the nuclei becomes dense. After pretreating the cells with 2.5 µg/mL and 5 µg/mL 17E6 and ab58524, respectively, Aβ-induced apoptosis was largely reduced in hippocampal neurons, and the number of TUNEL-positive cells decreased. Furthermore, the apoptotic rate of cells in the 17E6 group was lower than the ab58524 group.

**Figure 4:**
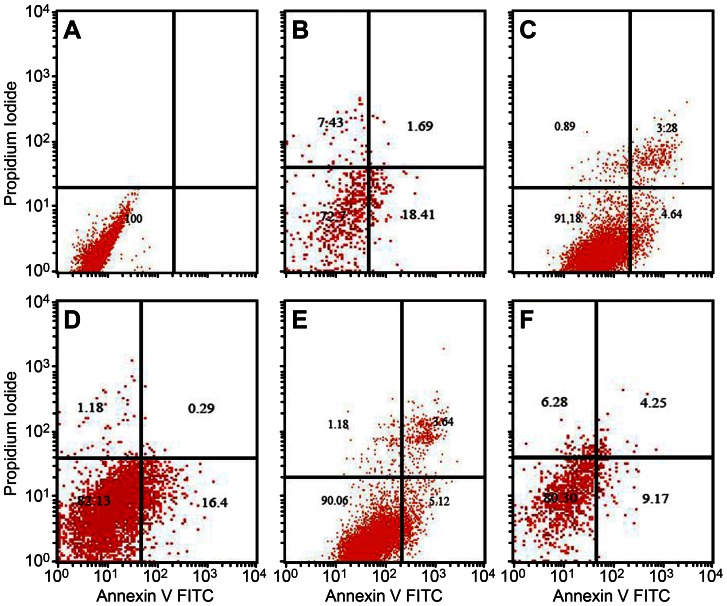
Detecting apoptosis in hippocampal neurons by flow cytometry. A: control group; B: Aβ treatment group; C: 17E6+ Aβ treatment group; D: 17E6+ Aβ+siFAK treatment group; E: ab58524+ Aβ treatment group; F: ab58524+ Aβ+siFAK treatment group.

In addition, we quantitatively analyzed changes in the ratio of the early apoptotic group and advanced mortality group in hippocampal neurons using Annexin V flow cytometry dually labeled with PI and EITC (Figure 4). The cells in the control group were all normal (100%). After treating the hippocampal neurons with Aβ, the apoptotic rate and mortality rate increased by 20.9%. When pre-treating the cells with 2.5 µg/mL 17E6 and 5 µg/mL ab58524, the Aβ-induced apoptotic rate and mortality rate in hippocampal neurons decreased by 7.92% and 8.76%, respectively.

### siFAK Lowers the Neuroprotective Effects of 17E6 and ab58524

After transfection and lentiviral-silencing of FAK, the MTT results showed that the survival rate decreased in the 17E6 and ab58524 groups (0.61±0.10 and 0.61±0.11, respectively) compared with the control cells (Figure 3). The siFAK groups showed statistically significant heterogeneity compared with the 17E6 and ab58524 groups (P<0.05).

Moreover, after transfection and lentiviral-silencing of FAK, the anti-apoptotic effects of 17E6 and ab58524 decreased, and the number of TUNEL-positive cells increased compared with the 17E6 and ab58524 groups as assessed by TUNEL staining (Figure 3). After DAPI staining, the nuclear stain in the siFAK group was enhanced compared with the 17E6 and ab58524 groups. Moreover, flow cytometry demonstrated similar patterns of change (Figure 4). After lentiviral siRNA FAK treatment, the apoptotic and mortality rate of the hippocampal neurons increased by 16.6% and 13.42%, respectively, compared with the 17E6 and ab58524 groups.

### Detection of FAK Gene Expression

**Figure 5:**
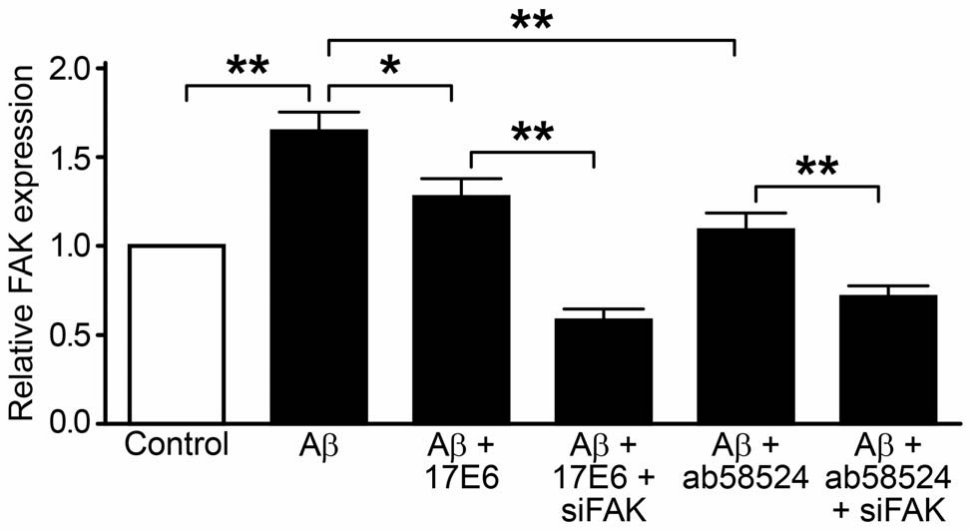
Real-time PCR detection of FAK gene expression; **P<0.01, *P<0.05.

A large number of studies have confirmed that integrins play an important role in AD pathology; however, it is still unclear whether FAK, a downstream component of the integrin pathway, participates in Aβ-induced apoptosis in hippocampal neurons. We used siRNA technology and real-time PCR to silence and detect FAK gene expression, respectively, in each experimental group. The cellular FAK gene expression in the Aβ treatment group was significantly increased compared with the control group (P<0.01), showing a 1.65±0.22-fold increase (Figure 5). After pretreatment with either 17E6 or ab58524, the FAK gene expression decreased compared with the Aβ treatment group but was still 1.23±0.20-fold and 1.09±0.19-fold higher compared with the control group, respectively. As previously described, the FAK gene expression was reduced after silencing with siRNA technology, which was 0.59±0.13-fold and 0.72±0.12-fold lower compared with the control group. There was also statistically significant heterogeneity compared with the 17E6 and ab58524 treatment groups (P<0.01).

### Detection of FAK and ρFAK Protein Expression

**Figure 6:**
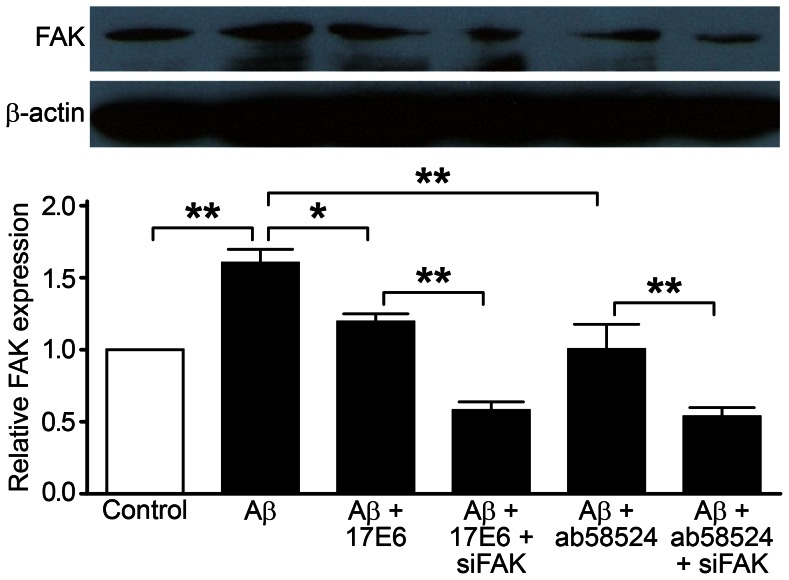
Western blot detection of FAK protein expression; **P<0.01, *P<0.05.

**Figure 7:**
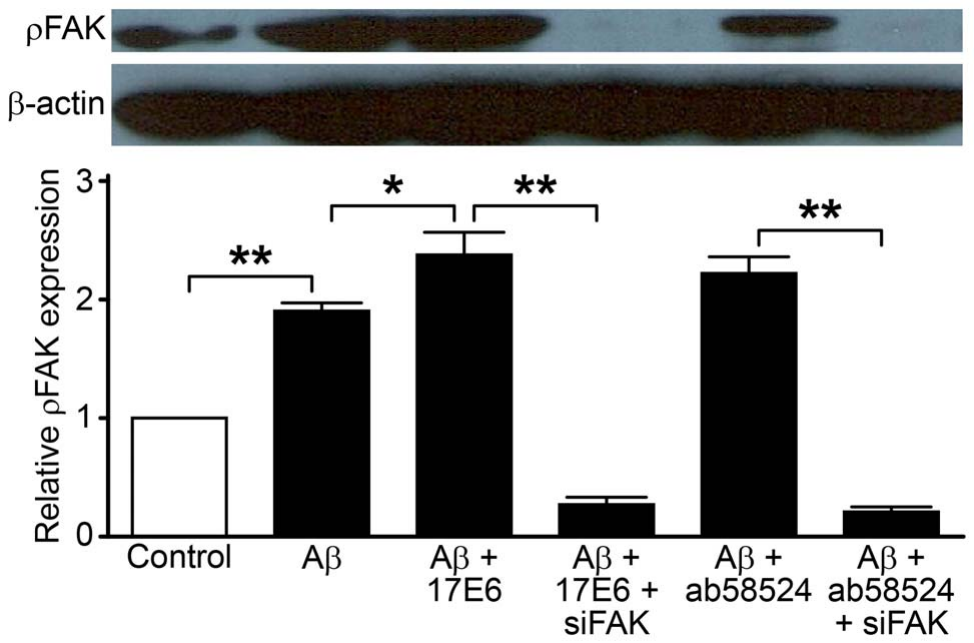
Western blot detection of ρFAK protein expression; **P<0.01, *P<0.05.

We used siRNA technology to silence FAK and Western blot to detect FAK protein and its phosphorylation status. The cellular FAK protein expression and phosphorylation status in the Aβ treatment group was significantly increased (1.60±0.21-fold and 1.90±0.16-fold, respectively) compared with the control group (P<0.01) (Figures 6 and 7). After pretreatment with 17E6 and ab58524, the FAK protein expression was reduced compared with the Aβ treatment group, which had values of 1.19±0.13-fold and 2.38±0.42-fold, respectively. There was statistically significant heterogeneity compared to the Aβ treatment groups (P<0.05 and P<0.01); however, the phosphorylation status, which was 2.38±0.42-fold and 2.22±0.32-fold, respectively, was reversed compared with the control group. In addition, there was statistically significant heterogeneity compared with the Aβ treatment groups (P<0.05), which corresponded to the final apoptotic changes. Furthermore, after lentiviral FAK-silencing, the 17E6 and ab58524 pretreatment groups were 0.58±0.13-fold and 0.53±0.14-fold lower compared with the control group, respectively, and the phosphorylated FAK status was 0.27±0.14-fold and 0.21±0.09-fold lower, respectively. Both groups were significantly reduced compared with the 17E6- and ab58524-pretreatment groups (P<0.01).

### Discussion and Conclusions

The Aβ hypothesis has been widely studied and supported by many studies in the pathogenesis of AD [7], [20]. Previous studies have shown that neurotoxicity generated by the abnormal production and accumulation of Aβ causes defects in the synaptic function and plasticity and directly leads to memory loss in the central nervous system at prophase [7], [21], [22]. At metaphase, neurotoxicity induces hyperplasia in microglia and astrocytes and generates extracellular nerve fiber tangles and intracellular senile plaques because of hyperphosphorylation of the Tau protein [23]. At anaphase, neurotoxicity results in the apoptosis and autophagy of neurons in the hippocampus and cortex, finally leading to dementia [4]. Aβ, which is secreted by brain tissue, transfers signals from several plasmalemma mediators from the outside to the inside of the cell, activates a succession of signaling pathways in cells, which results in physiological or pathological changes, including cell survival, proliferation, growth and apoptosis. Transmembrane heterodimer integrins, which are widely distributed on the cell membrane, are a very important type of transmembrane cellular adhesion molecule. Integrins mediate cell-cell and cell-extracellular matrix interactions. Several studies have shown that integrins have a close relationship with the growth of the central nervous system, cell cycle, synaptic function and memory formation. In addition, integrins are involved in very complex signaling pathways and pathogenesis [24], [25]. However, it is still unclear whether integrins contribute to the apoptosis of hippocampal neurons and participate in related signaling pathways.

In this study, we modeled Aβ-induced neurotoxicity in hippocampal neurons in vitro. According to our analyses on cellular activity and changes in cellular apoptosis, we have shown that both the specific antagonists 17E6 and ab58524 of αν and β1 integrins, respectively, inhibit the apoptosis of hippocampal neurons induced by Aβ. Conversely, we also demonstrated that both αν and β1 are involved in the apoptosis of hippocampal neurons induced by Aβ. Interestingly, although 17E6 and ab58524 increase the survival rate of neurons and protect neurons against apoptosis induced by Aβ, the survival rate of these neurons decreases compared with the control group. This evidence demonstrates that 17E6 and ab58524 may partially interrupt some of the apoptotic signaling pathways activated by Aβ. In addition to αν and β1, other integrin subunits or cellular adhesive molecules may also participate in the apoptotic process induced by Aβ in hippocampal neurons [26], [27].

FAK, which resides in the adhesive compomers formed by integrins located in the cell-extracellular matrix binding sites, is a significant downstream target protein in the integrin family and has several functions [28], [29]. FAK mediates the aggregation of Itgs and its multiple downstream signaling pathways, such as PI3K/Akt, tumor suppressor protein p53, Fyn, MAPK, ERK2 and GS3K, among others, which further regulate cell survival, apoptosis and cell proliferation [30], [31], [32], [33]. In this study, we demonstrated that following lentiviral-FAK silencing, the neuroprotective effects of 17E6 and ab58524 may by partially abolished, and the neuronal survival rate decreases as the apoptotic rate increases, demonstrating that FAK contributes to the Aβ-induced apoptosis mediated by αν and β1 in hippocampal neurons. We also showed that the neuroprotective effects of 17E6 and ab58524 were not completely abolished after siFAK treatment. The survival rate of cells significantly decreased as the apoptotic rate increased. All of these results indicate that other signaling pathways may participate to mediate αν and β1 Itg function. It has been reported by Wright S that α2β1 and ανβ1 facilitate the accumulation of Aβ and formation of the Aβ network by Pyk2 [17]. Sylvie M also reported that ανβ3 and ανβ5 regulate mitotic cell death via ILK (integrin-linked kinase) and RhoB signaling pathways, which participate in the radioresistance of glioma cells [34]. Moreover, Itgs can regulate cell survival and the cell cycle via PI3K/Akt or Rac/JNK signaling pathways, which are activated by Cdc42 [35].

In this study, we found that FAK expression and its phosphorylation increased following Aβ treatment, which indicated that Aβ-treatment may induce FAK expression and initiate autophosphorylation at the Tyr397 site [36], [37], [38]. After treating the cells with 17E6 and ab58524, the expression of FAK decreased, and its phosphorylation increased. Treatment with 17E6 and ab58524 may further promote FAK phosphorylation and activate cell survival via PI3K/Akt signaling [35] [39] inhibit the expression of the tumor suppressor protein p53 [40] [41] or activate FAK/paxillin/p130^CAS^ signaling pathways [42]. Following siFAK treatment, FAK phosphorylation may be reduced, thus resulting in an increase in apoptosis.

Taken together, both αν and β1 Itgs participate to mediate Aβ-induced apoptosis in hippocampal neurons. The underlying pathogenesis may be related to activation of tyrosine-phosphorylation by FAK. According to our study, inhibition of the interaction between Aβ and Itgs or the interruption of FAK activation may effectively inhibit apoptosis in hippocampal neurons in AD pathogenesis. Thus, this study may provide insight towards the development of novel AD treatments.

## Supporting Information

Figure S1 **MAP-2/DAPI double immunofluorescence examination of hippocampal neuronal purity (×400).**
(TIF)

Figure S2 ***Hpa*** **I/** ***Xho*** **I enzyme-digested pFU-GW-iRNA carriers.** 1: plasmids without enzyme digestion; 2: plasmids after *Hpa* I and *Xho* I enzyme-digested linearization; 3: DNA ladder marker: 10, 8, 6, 5, 4, 3.5, 3, 2.5, 2, 1.5 and 1 kb and 750, 500 and 250 bp.
(TIF)

Figure S3 **Agarose gel electrophoresis of three types of target sequences transfected with cloned PCR products.** 1: ddH_2_O control group; 2: empty vector control group; 3: DNA ladder marker: 5, 3, 2, 1.5 and 1 kb and 750, 500, 250 and 100 bp; 4∼8: colony group. A: S_1_ sequence carriers; B: S_2_ sequence carriers; C: S_3_ sequence carriers.
(TIF)

Figure S4 **GFP expression of viral stock solution (1 µL)-transfected 293T cells (×100).** A: S_1_ sequence carriers; B: S_2_ sequence carriers; C: S_3_ sequence carriers.
(TIF)

Figure S5 **Lentiviral transfection under different MOIs with or without polybrene (×200).**
(TIF)

Figure S6 FAK gene expression in transfected cells; ** ***P*** <0.01, * ***P*** **<0.05.**
(TIF)

Figure S7 FAK protein expression in transfected cells; * ***P*** **<0.05.**
(TIF)
