# Supplementary figures and images for: αν and β1 Integrins Mediate Aβ-Induced Neurotoxicity in Hippocampal Neurons via the FAK Signaling Pathway
Source: PLoS One. 2013 Jun 3;8(6):e64839. doi: 10.1371/journal.pone.0064839 (PMC3670848; doi:10.1371/journal.pone.0064839)

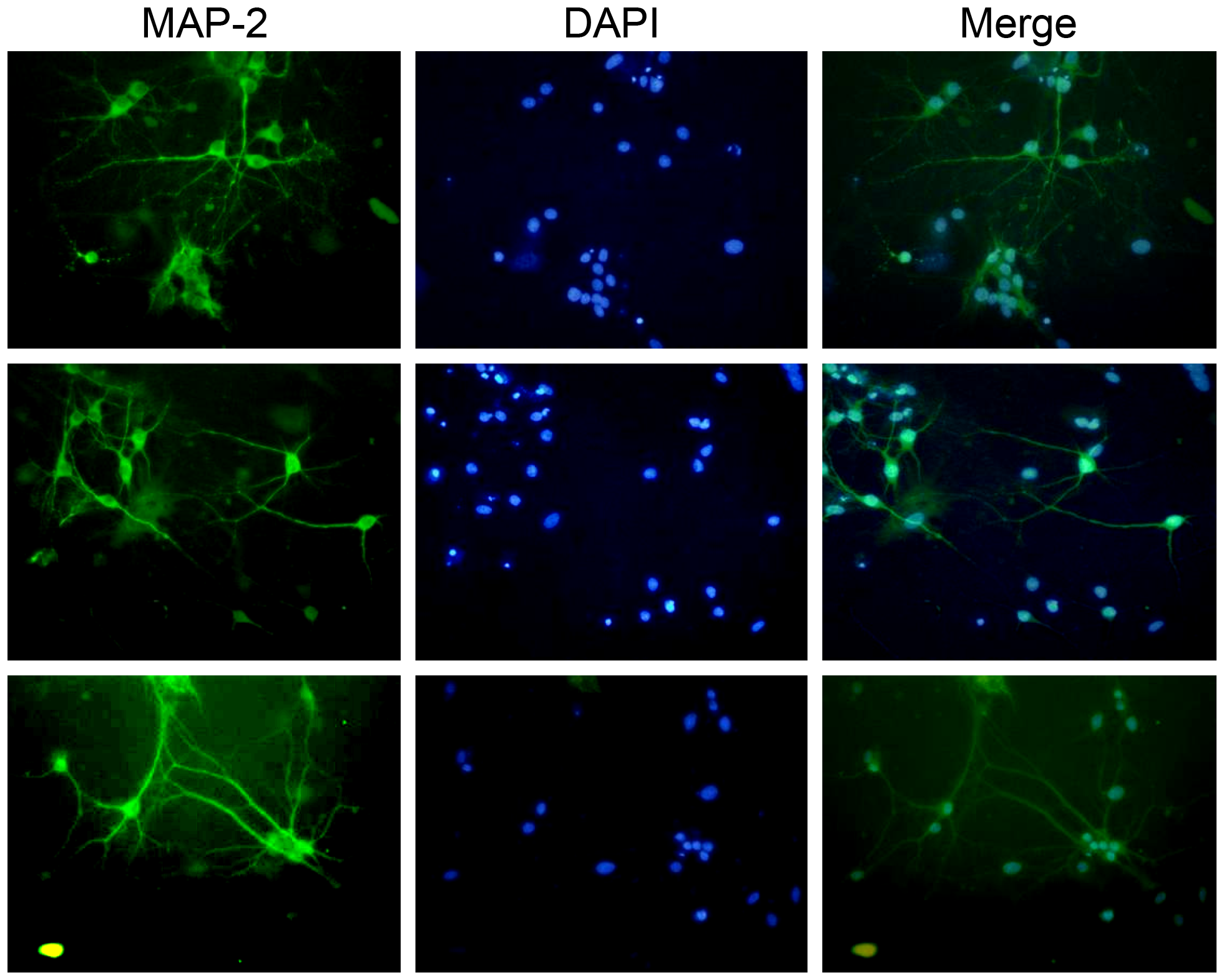

Supplement: Figure S1 — MAP-2/DAPI double immunofluorescence examination of hippocampal neuronal purity (×400). (TIF) [file pone.0064839.s001.tif]

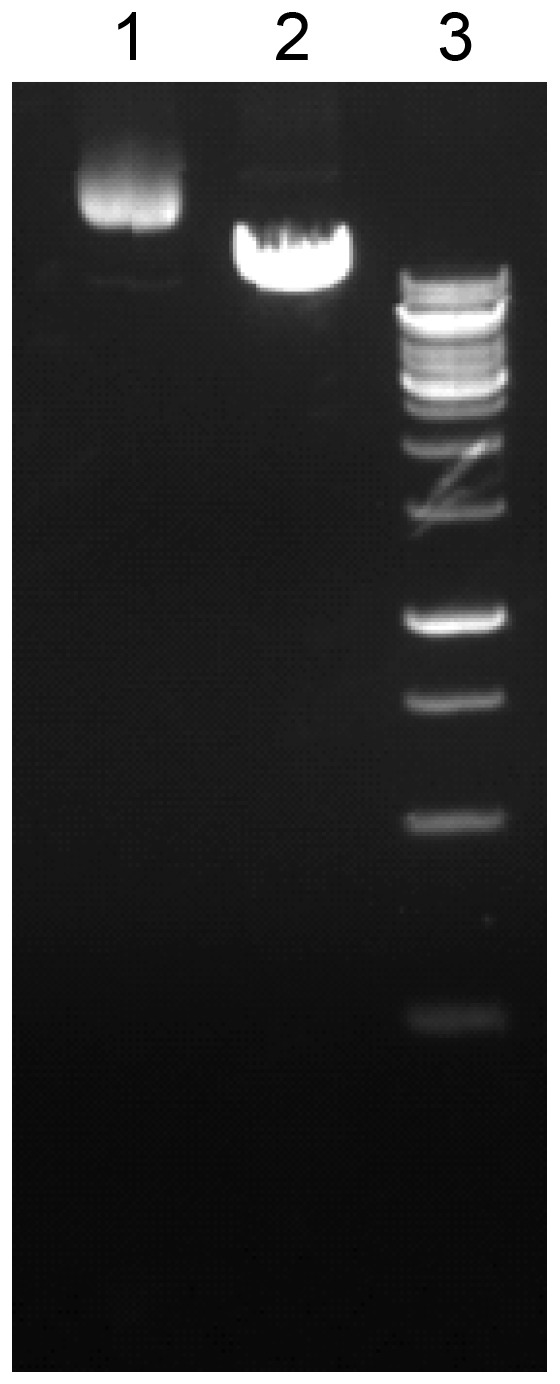

Supplement: Figure S2 — Hpa I/ Xho I enzyme-digested pFU-GW-iRNA carriers. 1: plasmids without enzyme digestion; 2: plasmids after Hpa I and Xho I enzyme-digested linearization; 3: DNA ladder marker: 10, 8, 6, 5, 4, 3.5, 3, 2.5, 2, 1.5 and 1 kb and 750, 500 and 250 bp. (TIF) [file pone.0064839.s002.tif]

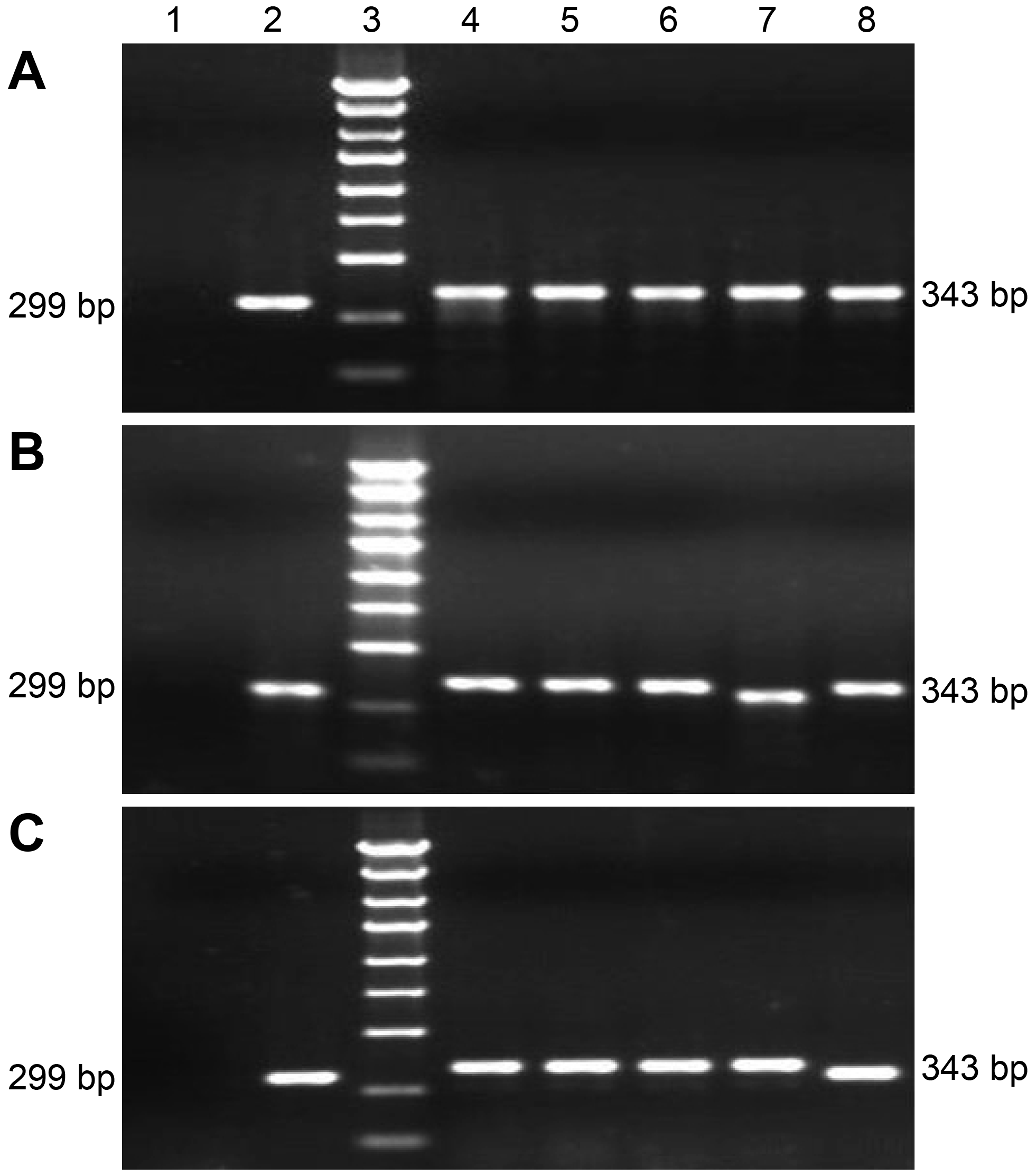

Supplement: Figure S3 — Agarose gel electrophoresis of three types of target sequences transfected with cloned PCR products. 1: ddH2O control group; 2: empty vector control group; 3: DNA ladder marker: 5, 3, 2, 1.5 and 1 kb and 750, 500, 250 and 100 bp; 4∼8: colony group. A: S1 sequence carriers; B: S2 sequence carriers; C: S3 sequence carriers. (TIF) [file pone.0064839.s003.tif]

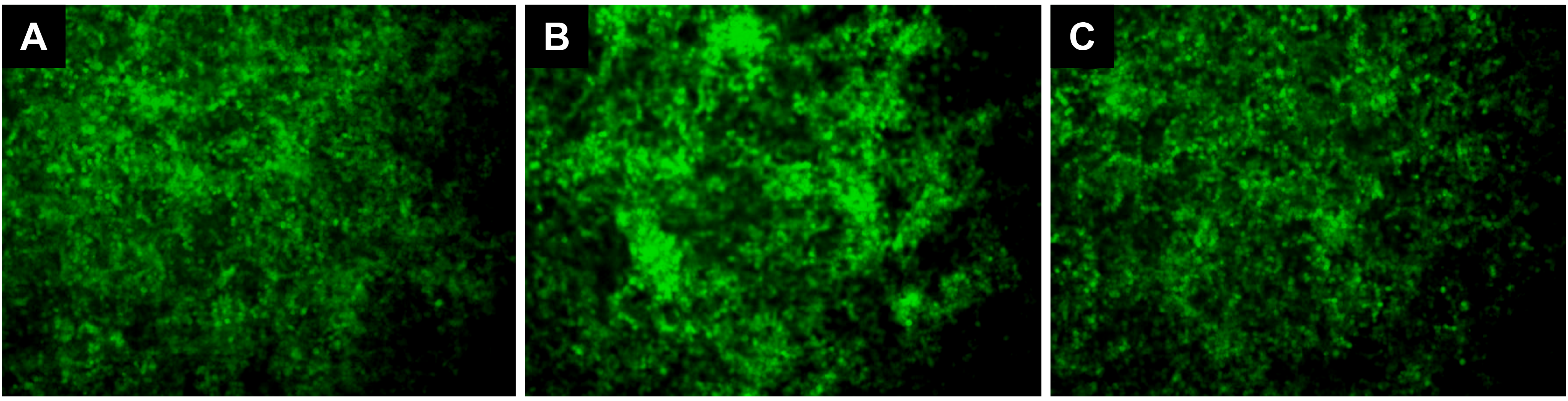

Supplement: Figure S4 — GFP expression of viral stock solution (1 µL)-transfected 293T cells (×100). A: S1 sequence carriers; B: S2 sequence carriers; C: S3 sequence carriers. (TIF) [file pone.0064839.s004.tif]

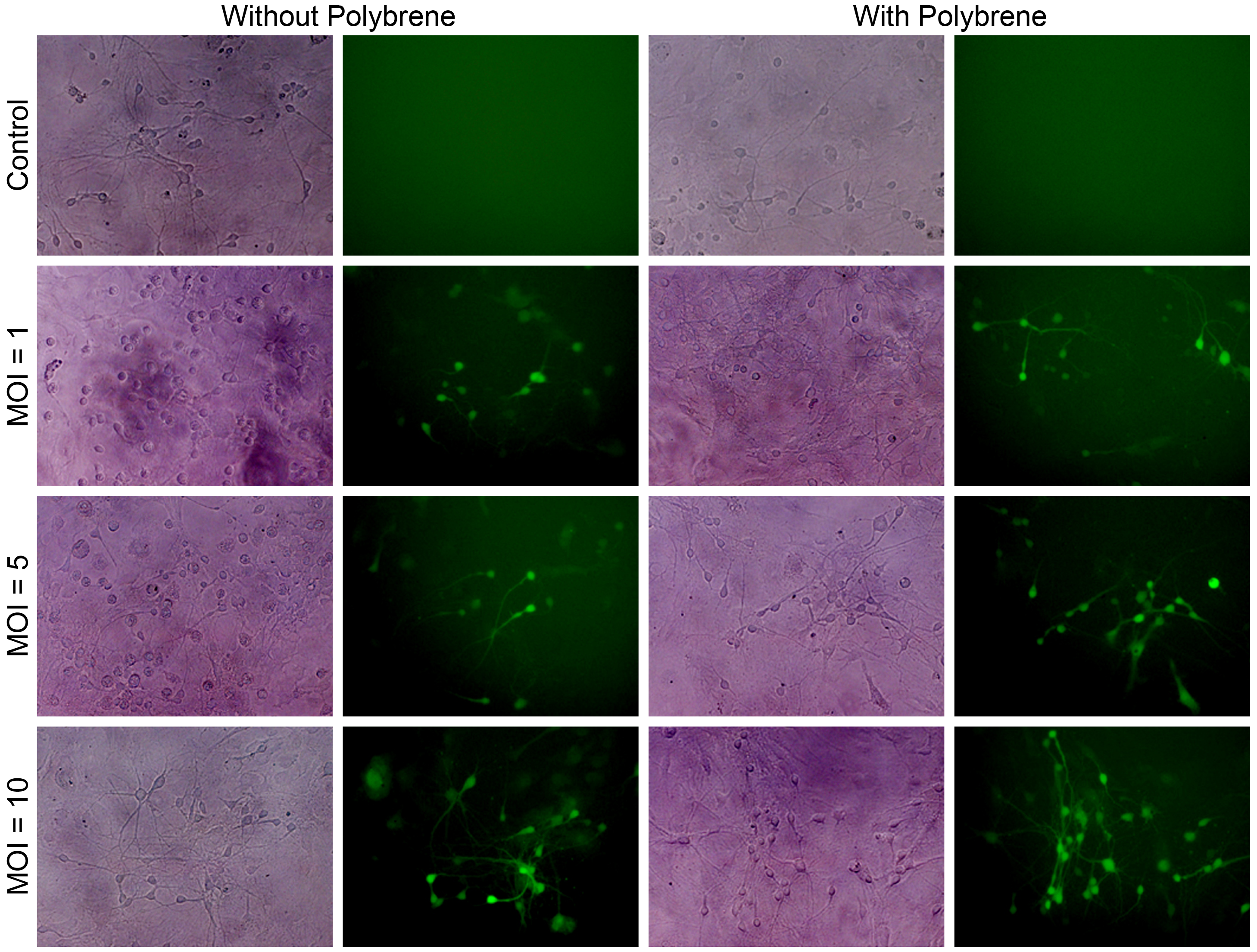

Supplement: Figure S5 — Lentiviral transfection under different MOIs with or without polybrene (×200). (TIF) [file pone.0064839.s005.tif]

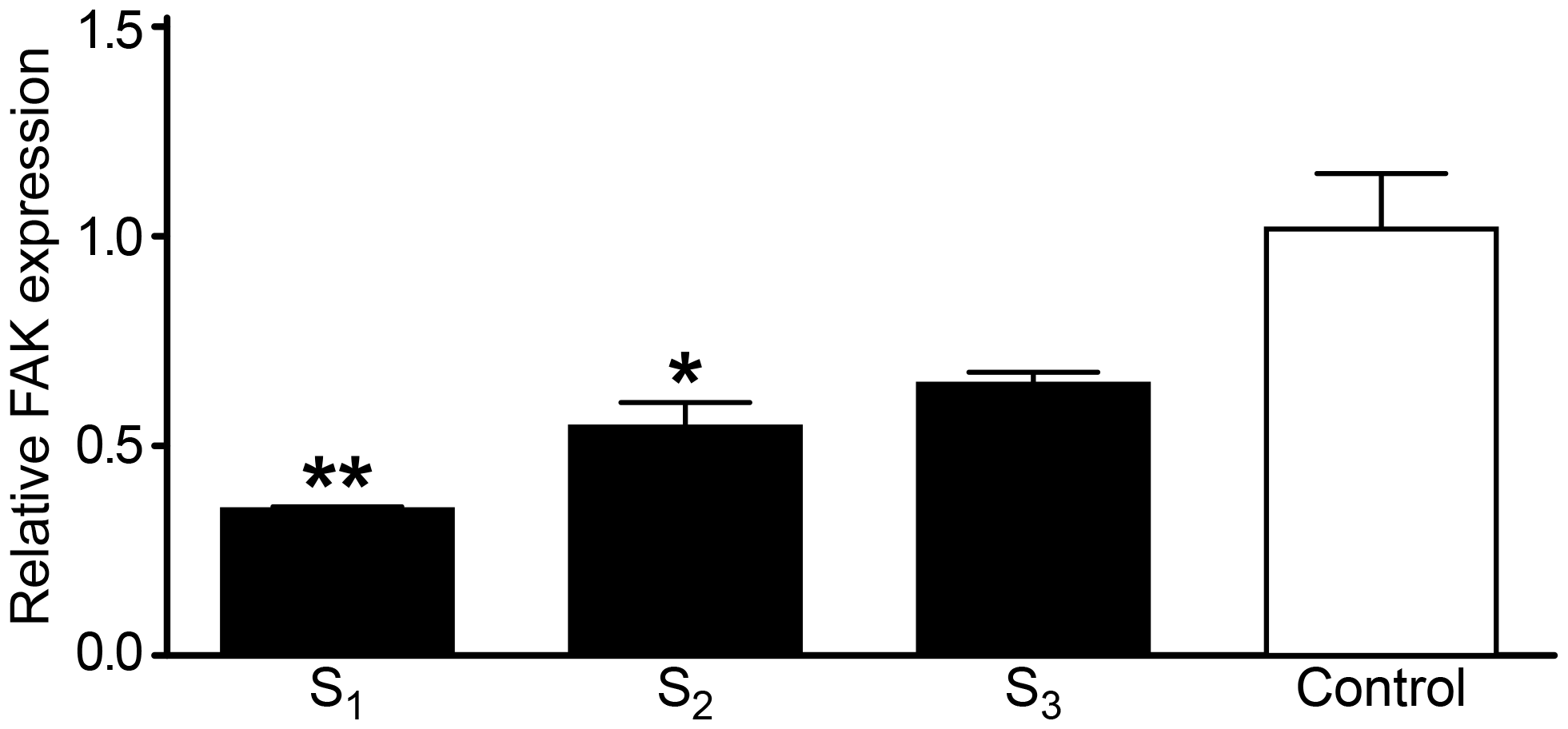

Supplement: Figure S6 — FAK gene expression in transfected cells; ** P <0.01, * P <0.05. (TIF) [file pone.0064839.s006.tif]

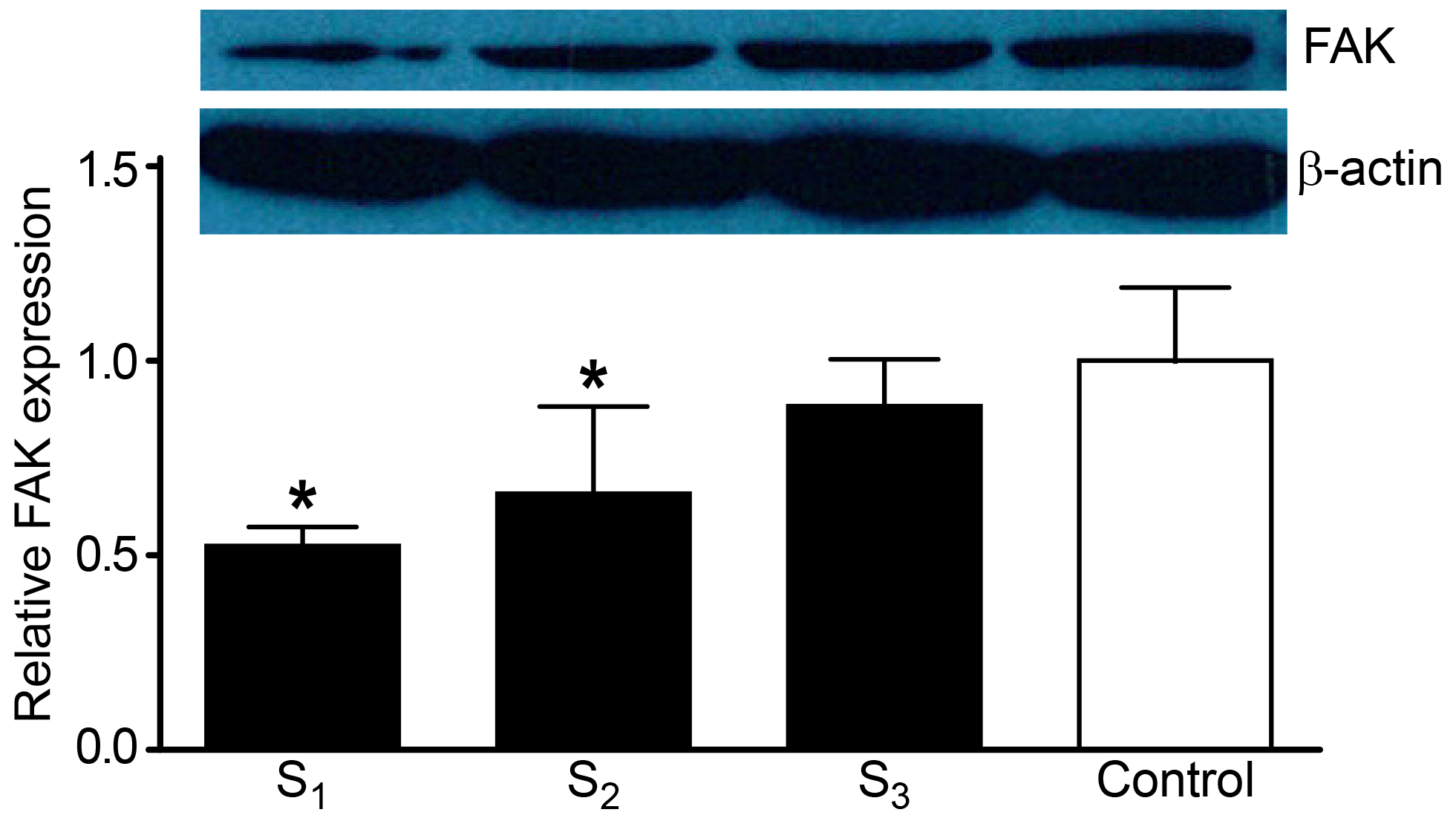

Supplement: Figure S7 — FAK protein expression in transfected cells; * P <0.05. (TIF) [file pone.0064839.s007.tif]
